# A survey of argasid ticks and tick-associated pathogens in the Peripheral Oases around Tarim Basin and the first record of *Argas japonicus* in Xinjiang, China

**DOI:** 10.1371/journal.pone.0208615

**Published:** 2018-12-26

**Authors:** Li Zhao, Xiang-Mei Lin, Fei Li, Kai-Rui Li, Bo He, Lu-Yao Zhang, Jiao-Jiao Pan, Qiang-Rong Wang, Jia-Min Gao, Nicholas Johnson, Xiang-Fen Yuan, Ji-Zhou Lv, Shao-Qiang Wu, Yong-Hong Liu

**Affiliations:** 1 College of Animal Science, Tarim University; Key Laboratory of Tarim Animal Husbandry Science and Technology of Xinjiang Production & Construction Corps, Alar, People’s Republic of China; 2 Institute of Animal Quarantine, Chinese Academy of Inspection and Quarantine, Beijing, People’s Republic of China; 3 Animal Loimia Controlling and Diagnostic Center of Aksu Region, People’s Republic of China; 4 Animal and Plant Health Agency, Woodham Lane, Surrey, United Kingdom; Onderstepoort Veterinary Institute, SOUTH AFRICA

## Abstract

Argasid ticks (Acari: Argasidae) carry and transmit a variety of pathogens of animals and humans, including viruses, bacteria and parasites. There are several studies reporting ixodid ticks (Acari: Ixodidae) and associated tick-borne pathogens in Xinjiang, China. However, little is known about the argasid ticks and argasid tick-associated pathogens in this area. In this study, a total of 3829 adult argasid ticks infesting livestock were collected at 12 sampling sites of 10 counties in the Peripheral Oases, which carry 90% of the livestock and humans population, around the Tarim Basin (southern Xinjiang) from 2013 to 2016. Tick specimens were identified to two species from different genera by morphology and sequences of mitochondrial 16S rRNA and 12S rRNA were derived to confirm the species designation. The results showed that the dominant argasid ticks infesting livestock in southern Xinjiang were *Ornithodoros lahorensis* (87.86%, 3364/3829). *Ornithodoros lahorensis* was distributed widely and were collected from 10 counties of southern Xinjiang. *Argas japonicus* was collected from Xinjiang for the first time. In addition, we screened these ticks for tick-associated pathogens and showed the presence of DNA sequences of *Rickettsia* spp. of Spotted fever group and *Anaplasma* spp. in the argasid ticks. This finding suggests the potential role for *Argas japonicus* as a vector of pathogens to livestock and humans.

## Introduction

Ticks could carry and transmit a variety of pathogens, including viruses, bacteria, rickettsiae, spirochetes, protozoans, chlamydia, mycoplasma and nematodes [1–4]. Two families of ticks are of medical significance: Ixodidae (hard ticks) and Argasidae (soft ticks). Argasid ticks comprise about 185 species from four genera. Approximately 19 species of Argasidae have been identified in China [5].

As the largest province of China, Xinjiang Uygur Autonomous Region covers over one-sixth of the country’s territory, including the majority of the arid areas in the country [6]. Forty two species of ticks in 9 genera have been identified in Xinjiang, which represent more than 1/3 of total tick species found in China [7]. Six species of argasid ticks from two genera have been reported in Xinjiang, including *Argas vespertilionis*, *Ar*. *persicus*, *Ar*. *reflexus*, *Ornithodoros lahorensis*, *Or*. *papillipes*, and *Or*. *tartakovskyi*.

The Tarim Basin is an endorheic basin, occupying an area of about 1,020,000 km^2^ [8]. It is sometimes used synonymously to refer to the southern half of the province, or southern Xinjiang. The vast territory, with complex geography, and diverse ecological environments within the Tarim Basin provides various habitats for argasid ticks [9]. Ninety percentage of humans and animals of Tarim Basin inhabit the Peripheral Oases, which are the most important ecosystems in the arid area [10]. Argasid ticks-livestock-humans interfaces in the Oases could promote the spread of tick-borne diseases.

More importantly, previous studies have shown that a high prevalence of tick-borne pathogens exists in Xinjiang province, such as piroplasms, *Anaplasma* spp., *Rickettsia* spp. and *Borrelia* spp. [6, 11–13]. However, these studies focused on ixodid ticks and there have been few research regarding argasid ticks and assciated tick-borne pathogens.

In the present study, the populations of argasid species associated with livestock at the sites around the Tarim Basin of Southern Xinjiang had been investigated. In addition, the presence of argasid tick-associated pathogens was assessed using molecular techniques.

## Materials and methods

### Sampling area, tick sampling and identification

**Fig 1:**
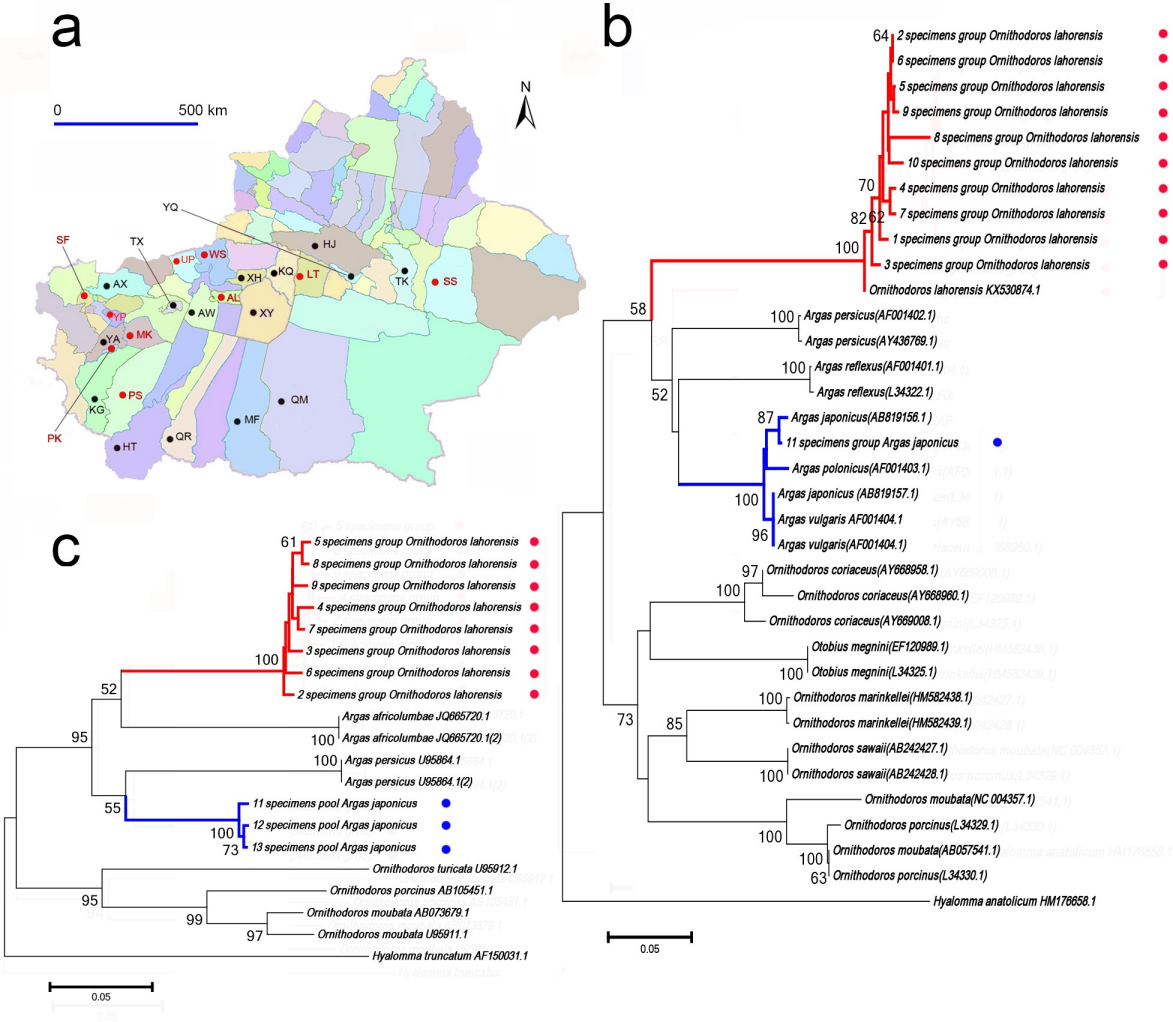
A map of Xinjiang Province and phylogenetic analyses of tick species based on 12S rRNA and 16S rRNA. A. argasid ticks were surveyed in 25 counties in southern Xinjiang. Argasid ticks were collected from 10 counties which are marked with red dots, while other 15 counties are marked with black dots, B. Neighbour joining phylogenetic analysis based on partial 16S rRNA sequences of ticks, C. Neighbour joining phylogenetic analysis based on partial 12S rRNA sequences of ticks. Bootstrap values are indicated at the nodes. Scale bar indicates the degree of divergence represented by a given length of branch. The red and blue dots indicate the DNA sequences of tick specimens acquired in this study. *Abbreviations*: AL, Aral; AX, Artux; AW, Awat; HJ, Hejing; HT, Hetan; KG, kargilik; KQ, Kuqa; LT, Luntai; MK, Makit; MF, Minfeng; PS, Pishan; PK, Poskam; QM, Qiemo; QR, Qira; SS, ShanShan; SF, Shufu; TX, Tumxuk; TK, Tuokexun; UP, Uqturpan; WS, Wensu; XY, Xayar; YP, Yopurga; XH, Xinhe; YQ, Yanqi; YA, Yarkand.

During the period from 2013 to 2016, we collected argasid tick specimens associated with livestock at twenty-seven sampling sites in Oases around the Tarim Basin of southern Xinjiang. This included farms at Aral, Artux, Awat, Hejing, Hetan, kargilik, Kuqa, Luntai, Makit, Minfeng, Pishan, Poskam, Qiemo, Qira, ShanShan, Shufu, Tumxuk, Tuokexun, Uqturpan, Wensu, Xayar, Xinhe, Yanqi, Yarkand, and Yopurga (Fig 1a). For the majority of counties, there was only one sampling site except Wensu county, which had three sampling sites.

Each sampling site was examined for argasid ticks associated with livestock from January to December during a one-year period at approximately 14 or 15-day intervals. Twenty-four samples were performed during the one-year period for each sampling site. The sampling sites of Uqturpan, Tumxuk, Pishan, Qiemo, and Tuokexun counties were examined in 2013. The sampling sites of Xinhe, Artux, Awat, Hejing, Hetan, and kargilik counties were examined in 2014. The sampling sites of Luntai, Kuqa, Qira, Yopurga, Minfeng, and Xayar counties were examined in 2015. The sampling sites of Yanqi, Aral, Makit, Poskam, Shufu, Shanshan, Wensu and Yarkand counties were examined in 2016.

Above counties are primarily livestock husbandry regions. The livestock reared in these regions are mainly autochthonous. The vast majorities (approximately 95%) of the sheep are Kazakh, Chinese Merino, Hu, and Altay breeds; the majority of the cattle are Xinjiang Brown, Holstein and Kazakh breeds.

Adult argasid ticks infesting animals were collected from body surface of livestock. Summary information regarding all of the collected specimens, including their location, host, number of ticks collected and the date of collection, was recorded (Table 1). Tick sampling was performed on the entire body (head, neck, feet, chest, shoulders, axilla, crotch, udder, abdomen, thorax, rib area, tail, perianal region and vulva) of each animal, including sheep and cattle on 12 farms of 10 counties. The collected ticks were kept alive until they were transferred to the laboratory. These ticks were counted, and identified to species by morphology according to standard morphological keys, including dorsal view and dorsal integument, and corroborated by deriving sequences for mitochondrial 16S ribosomal Ribonucleic Acid (rRNA) and 12S rRNA [14–17]. Ticks were grouped according to location/host of collection, date of collection, gender and species. All specimens are preserved in 70% ethanol. All of the livestock checked for ticks were grazed in the traditional manner, and there was no evidence that chemical acaricides were used prior to the tick sampling.

**Table 1:** The counts of each argasid species collected at the sampling sites around Tarim Basin between 2013 and 2016.

No. group of specimens	Hosts(No.)	*Ornithodoros lahorensis* (♂/♀)	*Argas japonicus* (♂/♀)	Collecting date	Counties
1	Cattle (2)	42(11/31)	/	Apr. 2016	Aral (AL)
2	Sheep (5)	263(162/101)	/	Mar. 2016	Makit (MK)
3	Sheep (5)	300(179/121)	/	Feb- Mar. 2016	Poskam (PK)
4	Sheep (2)	239(230/9)	/	Feb- Mar. 2016	Shufu (SF)
5	Sheep (7)	660(409/251)	/	Feb- Mar. 2016	Shanshan (SS)
6	Cattle (3)	594(0/594)	/	Feb- Mar. 2016	Wensu-1 (WS)
7	Cattle (3)	214(118/96)	/	Mar-Apr. 2015	LunTai (LT)
8	Sheep (5)	747(510/237)	/	Feb-Apr. 2015	Yopurga (YP)
9	Sheep (2)	203(122/81)	/	Apr. 2013	Uqturpan (UP)
10	Sheep (2)	102(0/102)	/	Apr. 2013	Pishan (PS)
11	Cattle (2)	/	109(0/109)	Feb- Mar. 2016	Wensu-2 (WS-2)
12	Cattle (3)	/	20(3/17)	Feb- Mar. 2016	Wensu-3 (WS-3)
13	Cattle (3)	/	336(271/65)	Feb- Mar. 2016	Wensu-1 (WS-1)
Total Number of ticks of specific species		3364(1741/1623)	465(274/191)		
Total Number of group of specimens of specific tick species		10	3		
Total number of ticks		3829(2015/1814)			
Total number of group of specimens		13			

### Nucleic acid extraction

Six representative tick specimens for each group of specimens were used for genomic DNA extraction. DNA templates were utilized for molecular identification of species and screening of pathogens.

Ethanol-preserved ticks were rinsed in distilled water and dried on filter paper. The total DNA was extracted using the DNeasy blood and tissue kit (Qiagen GmbH, Hilden, Germany) according to the manufacturer’s instructions. DNA was eluted in 80 μL elution buffer AE (provided with kit) and stored at -80°C until tested.

### Molecular identification of tick species

Partial sequences of 16S rRNA and 12S rRNA were amplified by PCR using the primers listed in S1 Table. PCR amplifications were performed using GoTaq G2 Flexi DNA polymerase (Promega, WI, USA). The reaction master mix was prepared according to the manufacture’s protocol and the PCR conditions described were used as published. PCR reaction mixtures consisted of: 1 μL of upstream primer (100 pmol/μL), 1 μL of downstream primers (100 pmol/μL), 1 μL PCR Nucleotide Mix (10 mM of each deoxynucleoside triphosphate), 6 μL Mgcl_2_ Solution (25mM/μL), 10 μL 5×PCR buffer, 0.25 μL Taq DNA polymerase (1 U/μL), 1 μL sample DNA template (50–100 ng/μL) and 29.75 μL Nuclease-Free Water for each PCR in a 50 μL mixture reaction.

All PCR reactions were performed with positive controls and negative controls. Total DNA (mitochondrial DNA included) extracted from *Hyalomma asiaticum asiaticum* was used as a positive control. Sterile water was included in the DNA extraction procedure and was used as a negative control. PCR amplifications products were electrophoresed on 1% agarose gels, stained with SYBR Green Safe DNA Gel Stain (Invitrogen, CA, USA) and visualized under ultra-violet light. DNA bands of the correct size were purified and sequenced by BGI Tech Solutions Co., LTD (Liuhe, Beijing) (S1 Table).

Representative sequences were submitted to Genbank. The 12S rRNA sequences of argasid ticks acquired in this study have been deposited in GenBank under accession numbers MG651960 to MG651967 (*Ornithodoros lahorensis*) and MG668793 to MG668795 (*Argas japonicus*). The 16S rRNA sequences of argasid ticks acquired in this study have been deposited in GenBank under accession numbers MG651950 to MG651959 (*Ornithodoros lahorensis*), and MH782636 (*Argas japonicus*). The detailed information is provided in S2 Table.

### Detection of DNA sequences of tick-associated pathogens

All primer sequences are provided in S1 Table and 78 argasid ticks of 13 groups of specimens were screened for tick-associated pathogens. Piroplasms were detected using the primer pair PIRO-A and PIRO-B, which targets 18S rRNA [18]. *Borrelia* spp. were detected using a primer pair that targeted the flagellin gene [19]. *Anaplasma* spp. were detected using two primer pairs that targeted major surface protein 4 gene (Msp4) and 16S rRNA [20, 21]. *Rickettsia* spp. was detected using a primer pairs targeting outer membrane protein B (ompB) protein [22, 23]. The summary of the pathogens found in the argasid ticks of southern Xinjiang is provided in Table 2.

**Table 2:** A summary of the pathogens found in the argasid ticks of southern Xinjiang.

Serial number	Pathogens	Tick species	No. group of specimens	Counties
1	*Candidatus Anaplasma boleense*	*Ornithodoros lahorensis*	7	LunTai (LT)
2	*Anaplasma ovis*	*Ornithodoros lahorensis*	9	Uqturpan (UP)
3	*Candidatus Anaplasma boleense*	*Argas japonicus*	11	Wensu-2 (WS-2)
4	*Rickettsia* spp. of spotted fever group	*Argas japonicus*	13	Wensu-1 (WS-1)

The master mix reagents were prepared according to the manufacture’s protocol and the PCR conditions described in previous studies were used. All PCR reactions were performed with positive controls and negative controls. Genomic DNA sequences of *Babesia divergens*, *Rickettsia* spp. (Spotted fever group), *Anaplasma phagocytophilum*, *Borrelia garinii* and *Coxiella burnetii* were used as positive controls. Sterile water was used as negative control. DNA bands of the correct size were purified and sequenced by BGI Tech Solutions (Liuhe, Beijing) Co., LTD. Representative sequences were submitted to Genbank (S2 Table).

### Sequences analysis

DNA sequences were assembled using Lasergene version 12.1 (DNASTAR) and edited in MEGA 5.0 [24, 25]. Sequence alignments were conducted using ClustalW within MEGA 5.0, using default parameters (open gap penalty = 10.0, extend gap penalty = 5.0) before subsequently being checked by visual inspection. Genetic distances were calculated based on the K2P model for all pair-wise comparisons in the matrix using MEGA5.0. Bootstrapping (1000 replicates) was utilized to estimate variance. Pairwise deletion was used for gaps/missing data. Based on K2P distances, phylogenetic trees were constructed with the combined data sets of major tick genera and tick-associated pathogens using the Neighbour-joining method as previous described [15, 26, 27].

## Results

### Tick collection and morphological identification

A total of 3829 adult argasid ticks, from two species of different genera, were collected at 12 sampling sites in 10 counties around the Tarim Basin of southern Xinjiang, including Wensu, Aral, Pishan, Makit, Shanshan, Poskam, Shufu, Luntai, Yopurga, Uqturpan counties (Fig 1a). Of these speimens, 2015 specimens were male while 1814 specimens were female.

The morphological characters of body dorsal and ventral views, dorsal integument, capitulum and legs of argasid ticks were used to species identification. Two argasid species, *Ornithodoros lahorensis* and *Argas japonicus*, were determined by the unique combination of morphological characters [28, 29].

Beside the morphological diagnosis, the phylogenetic analyses using sequences of 16S rRNA (Fig 1b) and 12S rRNA (Fig 1c) were performed. DNA sequences analyses showed that 16S rRNA sequence derived from group of specimens No.11 shares 99% sequence identity with 16S rRNA for *Argas japonicus* (AB819156.1 and AB819157.1) while 16S rRNA sequences derived from group of specimens No.1 to 10 share 99% sequence identity with 16S rRNA for *Ornithodoros lahorensis* (KX530874.1). Based on sampling sites, hosts, dates of collection, gender and species, we obtained 13 groups of specimens (Table 1). *Ornithodoros lahorensis* (87.86%, 3364/3829) was collected more frequently and was the widespread argasid species in the Tarim Basin while *Argas japonicus* (12.14%, 465/3829) was only collected from 3 farms of Wensu county. Furthermore, the seasonal population dynamics of *Ornithodoros lahorensis* and *Argas japonicus* were provided in S1 Fig.

### Detection of *Rickettsia* spp.

**Fig 2:**
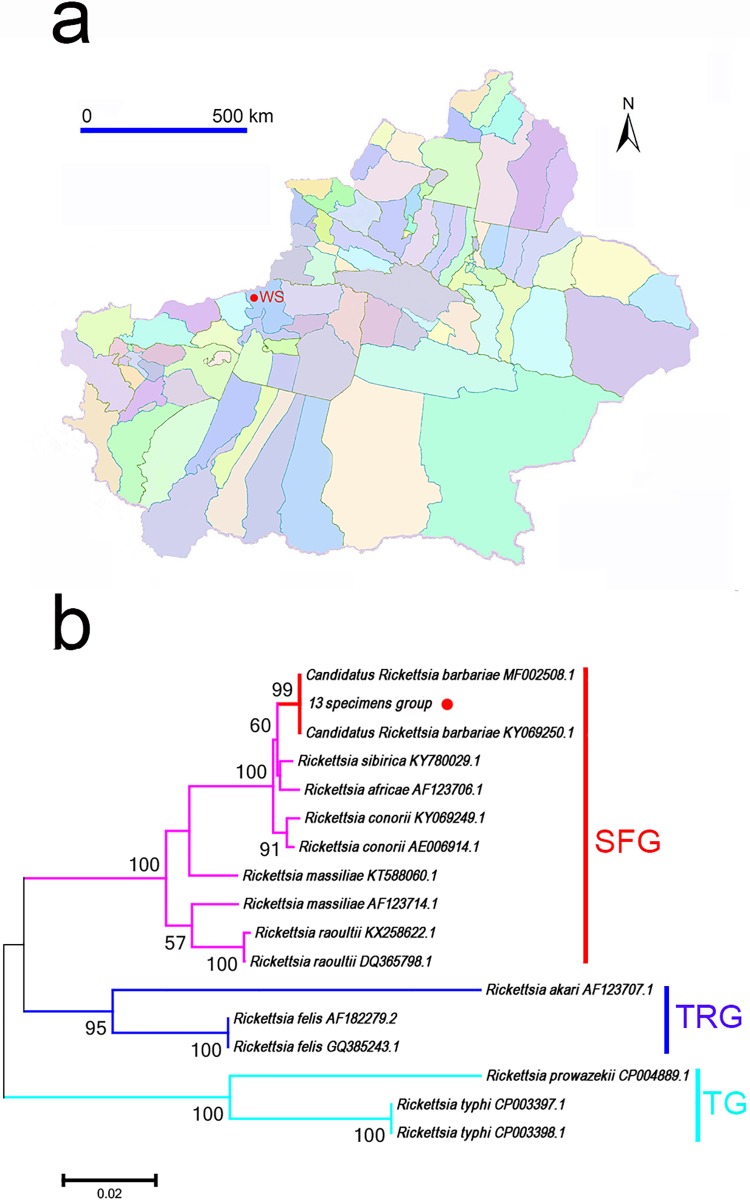
Detection and analysis of *Rickettsia* spp. in argasid ticks from southern Xinjiang. A. The sampling site of *Rickettsia* spp-positive tick is marked with red dot. B. Neighbour joining phylogenetic analysis based on partial ompB sequences of *Rickettsia* spp. Bootstrap values are indicated at the nodes. Scale bar indicates the degree of divergence represented by a given length of branch. The red dots indicate the sequences acquired in this study. SFG indicates the spotted fever group. TG indicates the typhus group. TRG indicates the transitional group of *Rickettisa* spp. *Abbreviations*: WS, Wensu.

DNA sequence of ompB of emerging *Rickettsia* spp. of spotted fever group (SFG) was detected in *Ar*. *japonicus* tick of group of specimens No.13, which was collected from Wensu county (Fig 2a). DNA sequence analysis showed that the sequence shares 99% sequence identity with the ompB of *Candidatus Rickettsia barbariae* (MF002508.1).

A phylogenetic analysis based on ompB of the representative *Rickettsia* spp. is shown in Fig 2b. The *Rickettsia* sequence derived from group of specimens No.13 clusters within the clade including *Rickettsia* species of SFG such as *Candidatus Rickettsia barbariae* (MF002508.1 and KY069250.1), *Rickettsia conorii* (AE006914.1) and *Rickettsia massiliae* (AF123714.1).

### Detection of *Anaplasma* spp.

**Fig 3:**
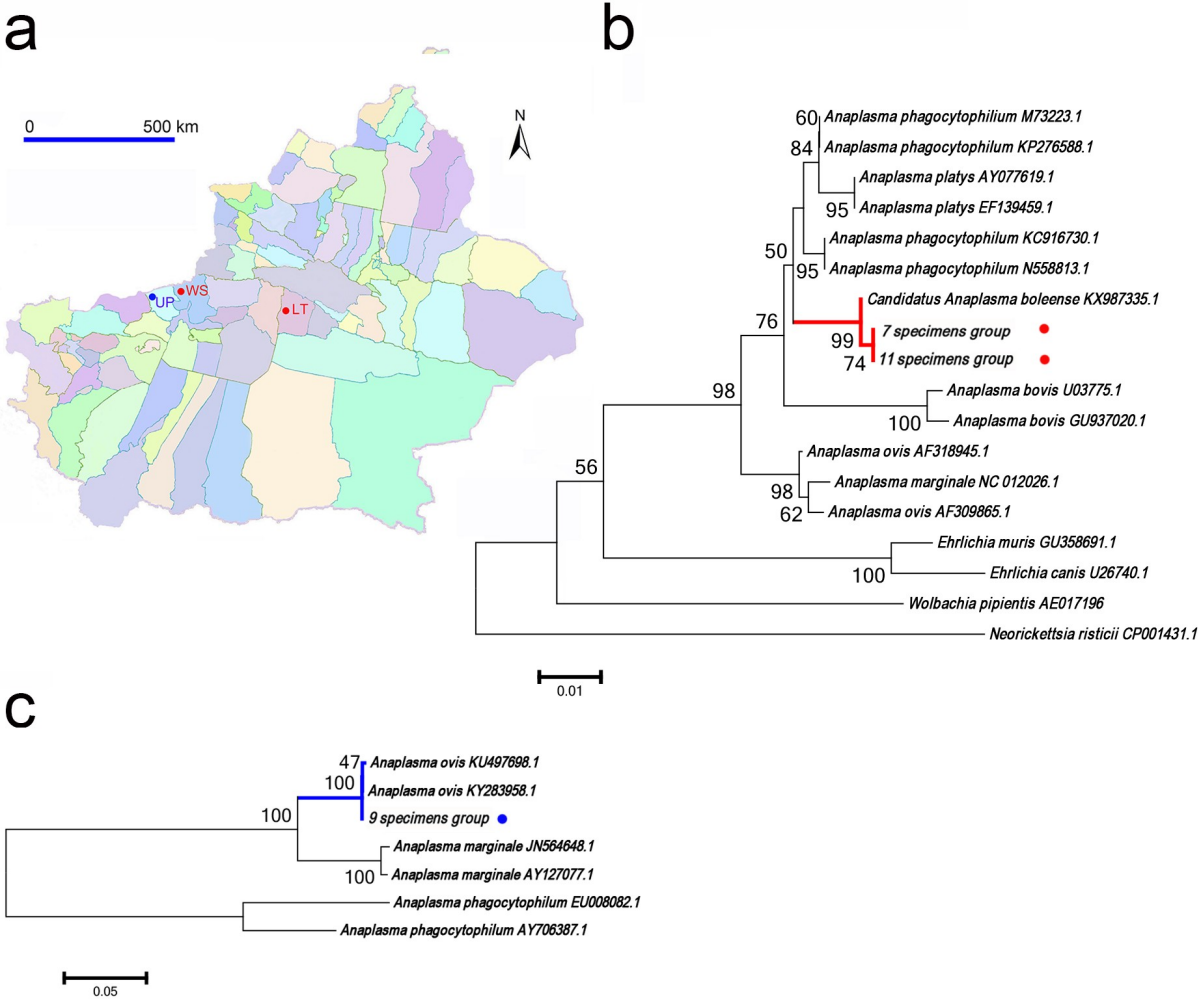
Detection and analysis of *Anaplasma* spp. in argasid ticks from southern Xinjiang. A. The sampling sites of *A*. *Boleense*-positive ticks are marked with red dots and the sampling site of *A*. *Ovis*-positive tick is marked with blue dot. B. Neighbour joining phylogenetic analysis based on partial 16S rRNA sequences of *Anaplasma* spp. C. Neighbour joining phylogenetic analysis based on partial msp4 sequences of *Anaplasma* spp. Bootstrap values are indicated at the nodes. Scale bar indicates the degree of divergence represented by a given length of branch. The red and blue dots indicate the sequences acquired in this study. *Abbreviations*: UP, Uqturpan; WS, Wensu; LT, Luntai.

DNA sequences of *Anaplasma* spp. were detected in 3 groups of specimens collected from LunTai, Uqturpan and Wensu counties (Fig 3a), composed of *Or*. *lahorensis* (group of specimens No.7, 9) and *Ar*. *Japonicus* (group of specimens No.11).

DNA sequence analysis of two amplicons derived from groups of specimens No.7 and 11 showed that they were identical to each other and share 99% sequence identity with 16S rRNA sequence of *Candidatus Anaplasma boleense* (KX987335.1). A phylogenetic tree based on 16S rRNA of the representative *Ehrlichia/anaplasma* spp. is shown in Fig 3b and both sequences acquired in this study cluster within the clade including *Candidatus A*. *boleense*. The *Candidatus A*. *boleense* associated tick specimens were identified as *Or*. *lahorensis* and *Ar*. *japonicus*. *Or*. *lahorensis* ticks were collected from Luntai county while *Ar*. *japonicus* ticks were collected from Wensu county. Above argasid ticks were removed from cattle.

The amplicon derived from group of specimens No.9 shared 99% sequence identity with msp4 of *A*. *ovis* (KY283958.1). The phylogenetic tree based on msp4 of the representative *Anaplasma* spp. is shown in Fig 3c and the sequence derived from group of specimens No. 9 clusters within the clade corresponding to *A*. *ovis*. The *A*. *ovis* associated tick specimens collected from Uqturpan county were identified as *Or*. *lahorensis*. Above *Or*. *lahorensis* ticks were removed from sheep.

### Detection of piroplasms, *Borrelia* spp. and *Coxiella burnetii*

The 78 argasid ticks were tested for the presence of DNA sequences of piroplasms, *Borrelia* spp. and *Coxiella burnetii*. All samples were negative for these pathogens.

## Discussion

As globally important arthropod vectors, tick infestations cause substantial blood loss from livestock and can transmit an extensive range of viral, bacterial and protozoan pathogens to vertebrate hosts [30, 31]. It has been reported that ixodid ticks and argasid ticks are widely distributed in Xinjiang and previous studies have focused on the ixodid ticks and associated tick-borne pathogens in this area [6, 7, 32]. However, little is known about argasid ticks and argasid tick-borne pathogens associated with the livestock sector and public health in Xinjiang.

In this study, two argasid tick species, *Or*. *lahorensis* and *Ar*. *japonicus*, were collected from livestock in southern Xinjiang. Only adult feeding argasid ticks were sampled. According to the seasonal population dynamics of argasid ticks (S1 Fig), *Ornithodoros lahorensis* ticks infesting livestock occurred from late February to early April while *Argas japonicus* infesting livestock occurred from February to March. Based on our research, these argasid ticks only occurred in spring rather than other seasons. We deduce that the seasonal argasid tick infestation of animals is mainly influenced by climatic factors such as temperature and humidity just as ixodid ticks. For the anatomical distribution on the animals, the majority of argasid ticks were collected from the abdomen, thorax and rib areas and the rest of ticks were collected from neck, crotch and udder areas. Several factors influence argasid tick preference for certain body areas, including greater blood flow, lower hair density and the animal’s inability to groom.

*Or*. *lahorensis*, collected from 10 counties, was the dominant argasid ticks infesting livestock in this area. *Or*. *lahorensis* is an important ectoparasite of livestock and wildlife in lowlands and mountains of many countries and regions including Tibet, Kashmir, and Southern former USSR to Saudi Arabia, Turkey, Iran, Greece, Bulgaria, and former Yugoslavia [33]. In the present study, we found that the geographical distribution of *Or*. *lahorensis* is nearly identical to the investigation conducted in Xinjiang three decades ago [34]. *Or*. *lahorensis* ticks were collected at sites in the northern and western rather than the southern and eastern parts of Tarim Basin. Moreover, the peak activity time of adult *Or*. *lahorensis* is also consistent with reports three decades ago. There is a substantial warming trend across Xinjiang province due to increased human activity and greenhouse gas emissions associated with animal production over the past 50 years [35]. Previous reports reveal that the most important factor to influence ticks may be host seeking tick activity periods and it has great effect on tick development rates and seasonal activity patterns [36]. We speculate that the stable herds in southern Xinjiang provide steady hosts for *Or*. *lahorensis* and host seeking activity of *Or*. *lahorensis* ticks do not change with warming environment during recent three decades.

*Argas japonicus* ticks were collected for the first time in Xinjiang province. The cattle, infested with *Ar*. *japonicus*, were born in Xinjiang and had never travelled outside the province. *Argas japonicus* was first collected from swallows and swallow nests in Japan and Korea [29]. Clinical investigations due to the cases of dermatitis caused by argasid tick infestation demonstrated that *Ar*. *japonicus* occurs in provinces of Northeastern China, including Jilin, Liaoning and Beijing [37]. These provinces are geographically close to Korea and Japan. The tranditional geographic range of *Ar*. *japonicus* seems to be restricted to the Northeastern Asia bordering the Pacific Ocean. This research confirms the presence of *Ar*. *japonicus* in Wensu county, which locates in the China-Kazakhstan-Kyrgyzstan border. The environment of central Asia is completely different from that of Northeastern Asia. It shows that *Ar*. *japonicus* may cover large areas as this species could adapt to sorts of environments. *Argas japonicus* is considered as a species mainly infesting wild birds and domestic poultry such as swallows and chickens and is found to occasionally attack humans. Our data expands the list of known natural hosts of *Ar*. *japonicus* ticks, adding cattle and sheep. In some farms of southern Xinjiang, the livestock are mixed cultured with poultry such as chickens and pigeons. We deduce that the poultry should be the original hosts for *Ar*. *japonicus* while the sheep and cattle are only occasional hosts. The livestock could be contracted by close contact cultured with tick-infested poultry. *Ar*. *japonicus* is of high medical significance as the tick-borne pathogens could be transmitted among birds, livestock and humans.

Ticks are considered to be one of the most important vectors of pathogens of medical and veterinary interest [3, 5]. Previous studies have demonstrated that *Or*. *lahorensis* is an important tick species infesting livestock and could transmit pathogens such as *Rickettsia* spp., *Anaplasma* spp. and piroplasms [33, 34]. Six representative ticks from each group of specimens were screened for pathogens and finally 78 of the 3829 ticks were examined in this study. The argasid ticks of the same species collected from one farm (the same group of specimens) are likely to belong to the similar population and the differences within one tick population in tick-borne pathogens would be limited. Therefore, there would be great chance to detect pathogens when six ticks per group of specimens were screened though the possibility of missed detection could not be avoided completely.

We detected DNA sequences of *Candidatus A*. *boleense* and *A*. *ovis* in *Or*. *lahorensis* ticks and this suggested the potential role for *Or*. *lahorensis* as a vector of *Anaplasma* spp. A case of human dermatitis as a result of a bite by *Ar*. *japonicus* was recorded in 1986, but no pathogens associated with *Ar*. *japonicus* was revealed until now [37]. For the first time, we described the *Ar*. *japonicus* associated pathogens and the DNA sequences of *Rickettsia* spp. of SPF and *Candidatus A*. *boleense* were detected. Tick borne *Rickettsia* spp. within SPF are associated with several human diseases such as Mediterranean spotted fever and Israeli spotted fever [38, 39]. *Ar*. *japonicus* could attack humans and *Ar*. *japonicus* associated *Rickettsia* spp. within SPF have important medical and biological significance.

The screening of pathogens was performed with fed argasid ticks and there was a possibility of acquiring the infection by blood meal from host carrier. In this study, pathogens were detected in argasid ticks collected from 4 farms. The herd sizes of these four farms were small and varied from 2 to 3. It was guaranteed that two or three specimens from the same host were screened. It was noticed that only one of the specimens from each group of specimens was positive for specific pathogen while other specimens were negative. If the origin of pathogens was from the carrier host blood meal, all the specimens from the same host should be positive for this pathogen. Based on the above phenomenon, we deduced that the origin of pathogens should be ticks themselves rather than host blood meal. However, the possibility of artificial tick-pathogens association could not be completely dismissed. Further research utilizing unfed argasid ticks is needed to accurately determine the role for argasid ticks as vectors of *Anaplasma* spp. and *Rickettsia* spp.

## Conclusions

In this study, a total of 3829 adult argasid ticks, *Or*. *lahorensis* and *Ar*. *japonicus*, were collected at 12 sampling sites in 10 counties around Tarim basin. The results show that at present *Or*. *lahorensis* is the dominant argasid species infesting livestock in southern Xinjiang and *Ar*. *japonicus* was first recorded in Xinjiang of China. Besides, DNA sequences of some tick-associated pathogens such as *Rickettsia* spp. of SFG, *Anaplasma* spp. were detected in argasid ticks of southern Xinjiang. The potential role of *Ar*. *Japonicus* as the vector of pathogens was described for the first time.

## Declarations

Permission was obtained from the farm owners before collection of the specimens.

## Supporting information

S1 Fig Seasonal population dynamics of argasid ticks associated with livestock during a one-year period (A, *Ornithodoros lahorensis*; B, *Argas japonicus*). (TIF)

S1 Table Primers used for identification of tick species and screening of pathogens. (DOC)

S2 Table Genbank Accession numbers for sequences of ticks and tick-borne pathogens. (DOC)
