# Supplementary figures and images for: A survey of argasid ticks and tick-associated pathogens in the Peripheral Oases around Tarim Basin and the first record of Argas japonicus in Xinjiang, China
Source: PLoS One. 2018 Dec 26;13(12):e0208615. doi: 10.1371/journal.pone.0208615 (PMC6306169; doi:10.1371/journal.pone.0208615)

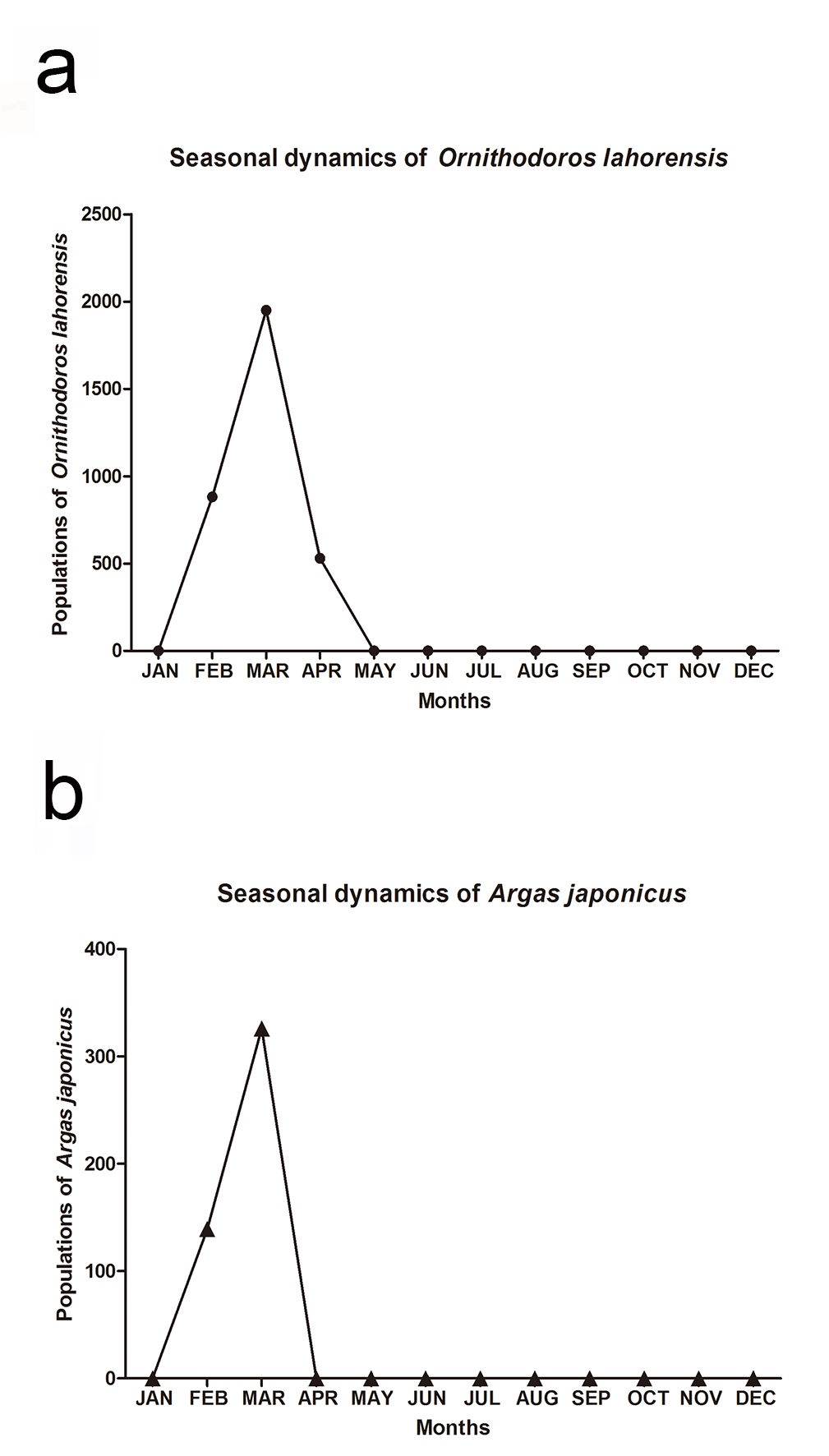

Supplement: S1 Fig — (TIF) [file pone.0208615.s001.tif]
