# Supplementary material for: A survey of argasid ticks and tick-associated pathogens in the Peripheral Oases around Tarim Basin and the first record of Argas japonicus in Xinjiang, China
Source: PLoS One. 2018 Dec 26;13(12):e0208615. doi: 10.1371/journal.pone.0208615 (PMC6306169; doi:10.1371/journal.pone.0208615)
Supplement: S1 Table — (DOC) [file pone.0208615.s002.doc]

**S1 Table Primers used for tick species identification and pathogens screening.**

| **Target** | **Gene Target** | **Primer pairs** | **Amplicon(bp)** |
| --- | --- | --- | --- |
| Arthropod species | 12S rRNA | 12S-F: 5'-AAACTAGGATTAGATACCCT-3',  12S-R: 5'-AATGAGAGCGACGGGCGATGT-3' | 320 |
| Arthropod species | 16S rRNA | 16S-F: 5'-TTAAATTGCTGTRGTATT-3',  16S-R: 5'-CCGGTCTGAACTCASAWC-3' | 455 |
| Piroplasma spp | 18S rRNA | Piro-a: 5'-AATACCCAATCCTGACACAGGG-3,  Piro-b: 5'-TTAAATACGAATGCCCCCAAC-3 | 426 |
| *Borrelia* spp | GroEL | Gro-F: 5'-TACGATTTCTTATGTTGAGGG-3′,  Gro-R: 5'-CATTGCTTTTCGTCTATCACC-3′ | 310 |
| *Anaplasma* spp. | Msp4 | MSP4-F: 5′-GGGAGCTCCTATGAATTACAGAGAATTGTTTAC-3′,  MSP4-R: 5′-CCGGATCCTTAGCTGAACAGGAATCTTGC-3′ | 867 |
|  | 16S rRNA | A16S-F: 5′-GCTGAATGTGGGGATAATTTAT-3′,  A16S-R: 5′-ATGGCTGCTTCCTTTCGGTTA-3′ | 641 |
| *Rickettsia* spp. | OmpB | OmpB-F: 5′-TACTTCCGGTTACAGCAAAGT-3′,  OmpB-R: 5′-AAACAATAATCAAGGTACTGT-3′ | 812 |
