# Supplementary material for: A survey of argasid ticks and tick-associated pathogens in the Peripheral Oases around Tarim Basin and the first record of Argas japonicus in Xinjiang, China
Source: PLoS One. 2018 Dec 26;13(12):e0208615. doi: 10.1371/journal.pone.0208615 (PMC6306169; doi:10.1371/journal.pone.0208615)
Supplement: S2 Table — (DOC) [file pone.0208615.s003.doc]

**S2 Table Genbank Accession numbers for sequences of ticks and tick-borne pathogens.**

| Gene | No. specimens group | Species | Accession Numbers |
| --- | --- | --- | --- |
| 12S rRNA ( Argasidae ) | 2# | *Ornithodoros lahorensis* | MG651960 |
|  | 3# | *Ornithodoros lahorensis* | MG651961 |
|  | 4# | *Ornithodoros lahorensis* | MG651962 |
|  | 5# | *Ornithodoros lahorensis* | MG651963 |
|  | 6# | *Ornithodoros lahorensis* | MG651964 |
|  | 7# | *Ornithodoros lahorensis* | MG651965 |
|  | 8# | *Ornithodoros lahorensis* | MG651966 |
|  | 9# | *Ornithodoros lahorensis* | MG651967 |
|  | 11# | *Argas japonicus* | MG668793 |
|  | 12# | *Argas japonicus* | MG668794 |
|  | 13# | *Argas japonicus* | MG668795 |
| 16S rRNA ( Argasidae ) | 1# | *Ornithodoros lahorensis* | MG651950 |
|  | 2# | *Ornithodoros lahorensis* | MG651951 |
|  | 3# | *Ornithodoros lahorensis* | MG651952 |
|  | 4# | *Ornithodoros lahorensis* | MG651953 |
|  | 5# | *Ornithodoros lahorensis* | MG651954 |
|  | 6# | *Ornithodoros lahorensis* | MG651955 |
|  | 7# | *Ornithodoros lahorensis* | MG651956 |
|  | 8# | *Ornithodoros lahorensis* | MG651957 |
|  | 9# | *Ornithodoros lahorensis* | MG651958 |
|  | 10# | *Ornithodoros lahorensis* | MG651959 |
|  | 11# | *Argas japonicus* | MH782636 |
| Msp4 (*Anaplasma*) | 9# | *Anaplasma ovis* | MG668814 |
| 16S rRNA (*Anaplasma*) | 7# | *Anaplasma boleense* | MG668800 |
|  | 11# | *Anaplasma boleense* | MG668811 |
| ompB (*Rickettsia*) | 13# | *Rickettsia* spp | MG668836 |
